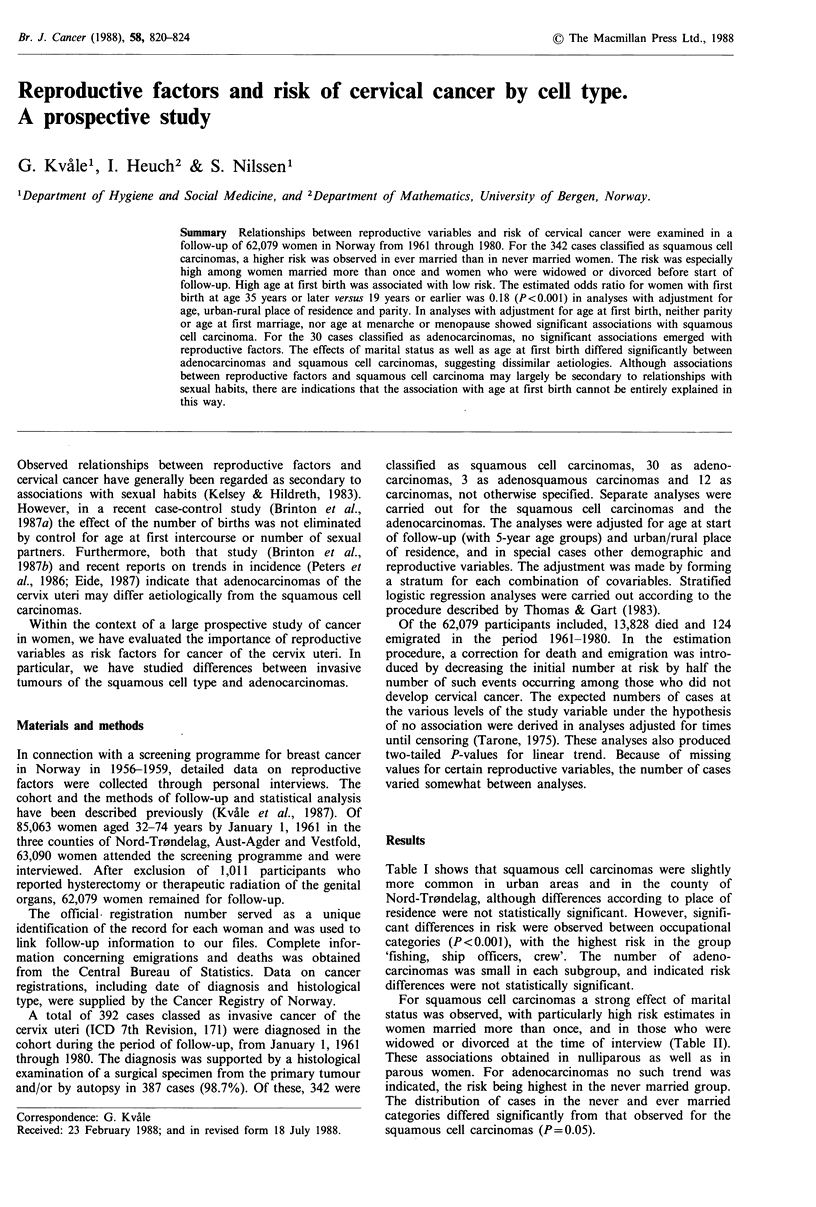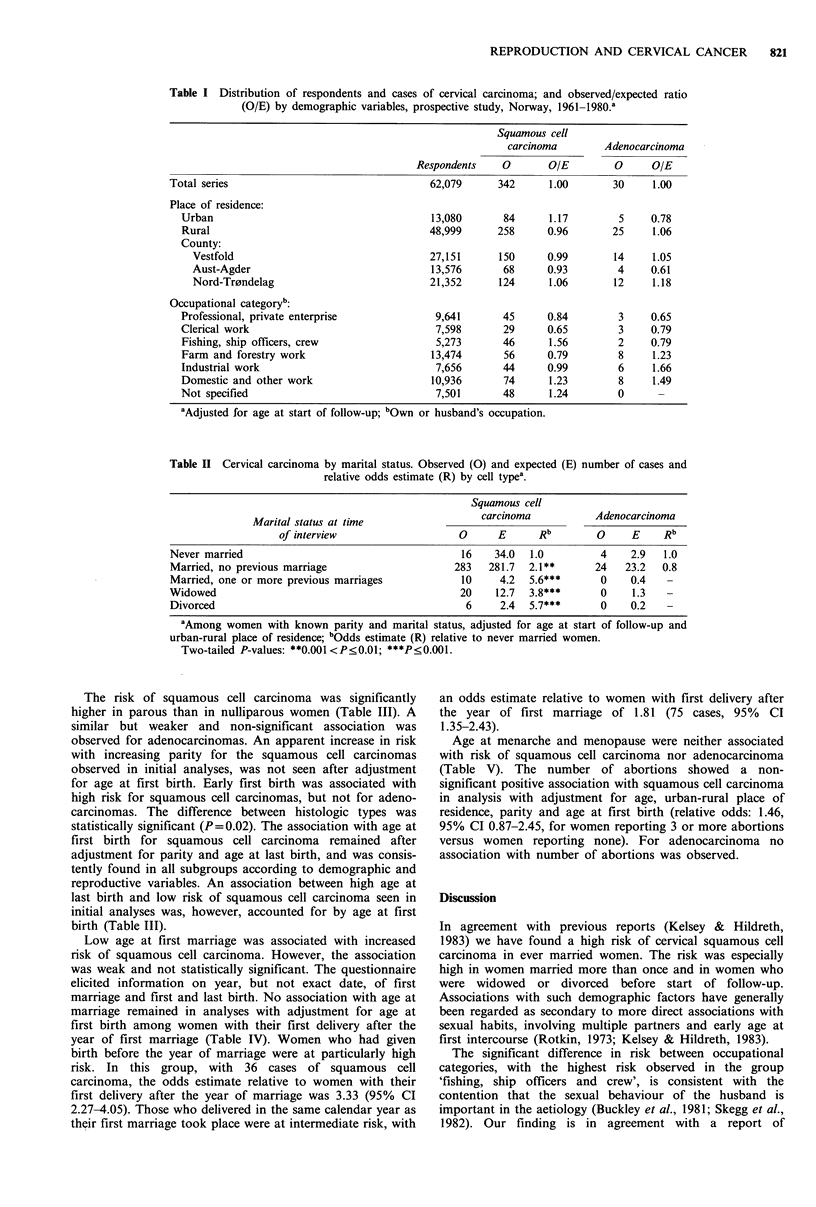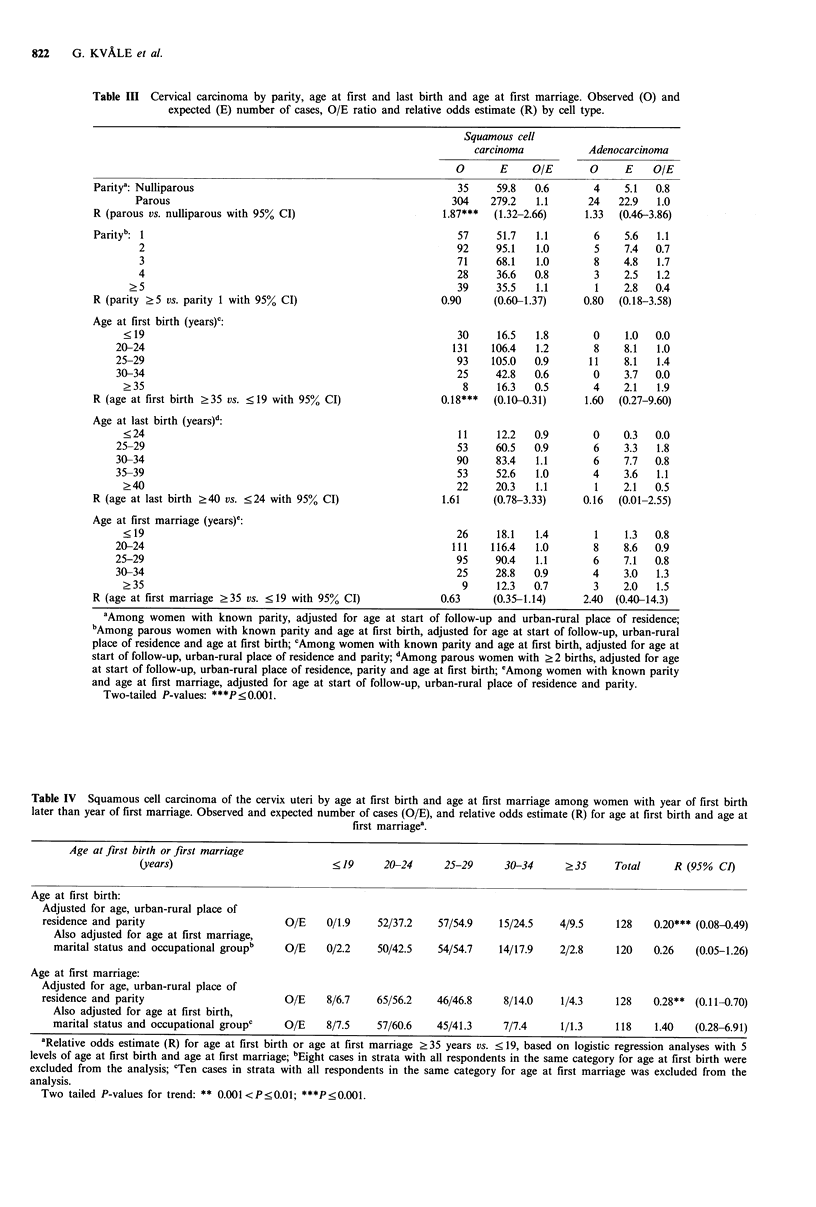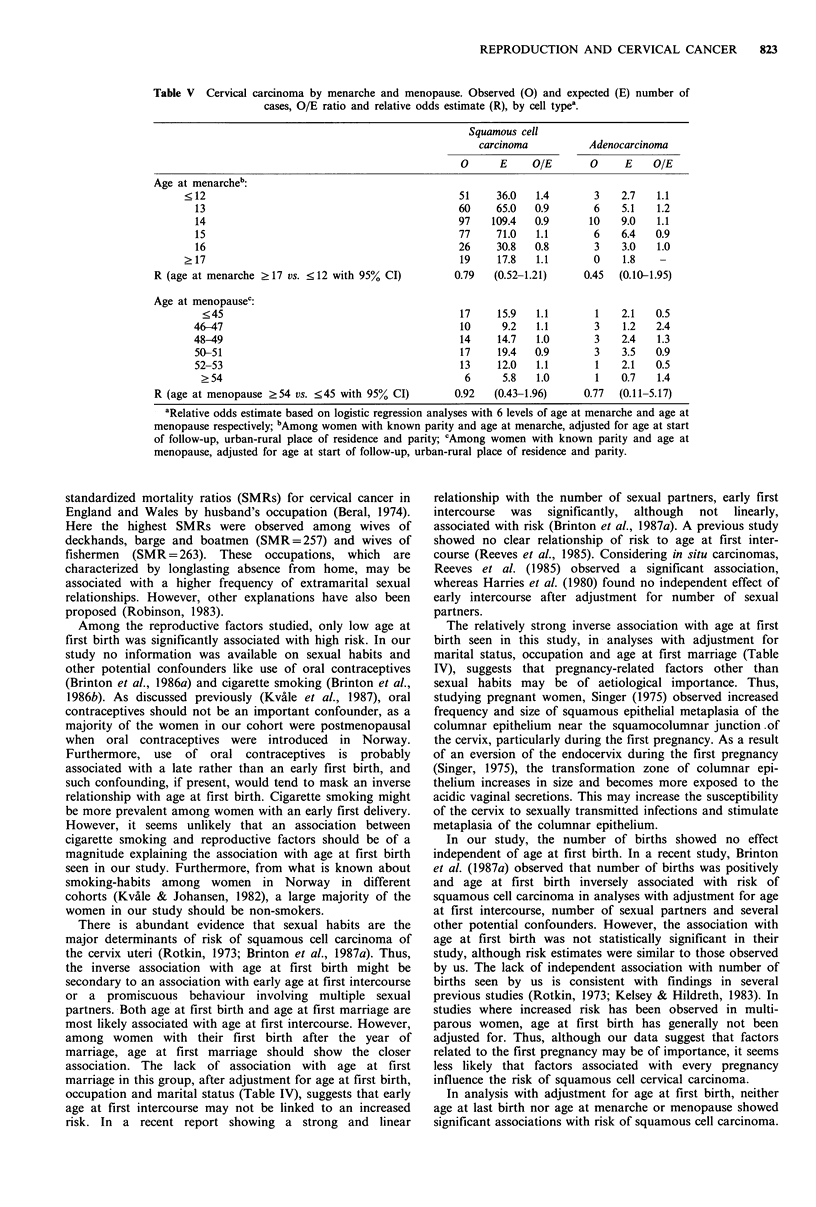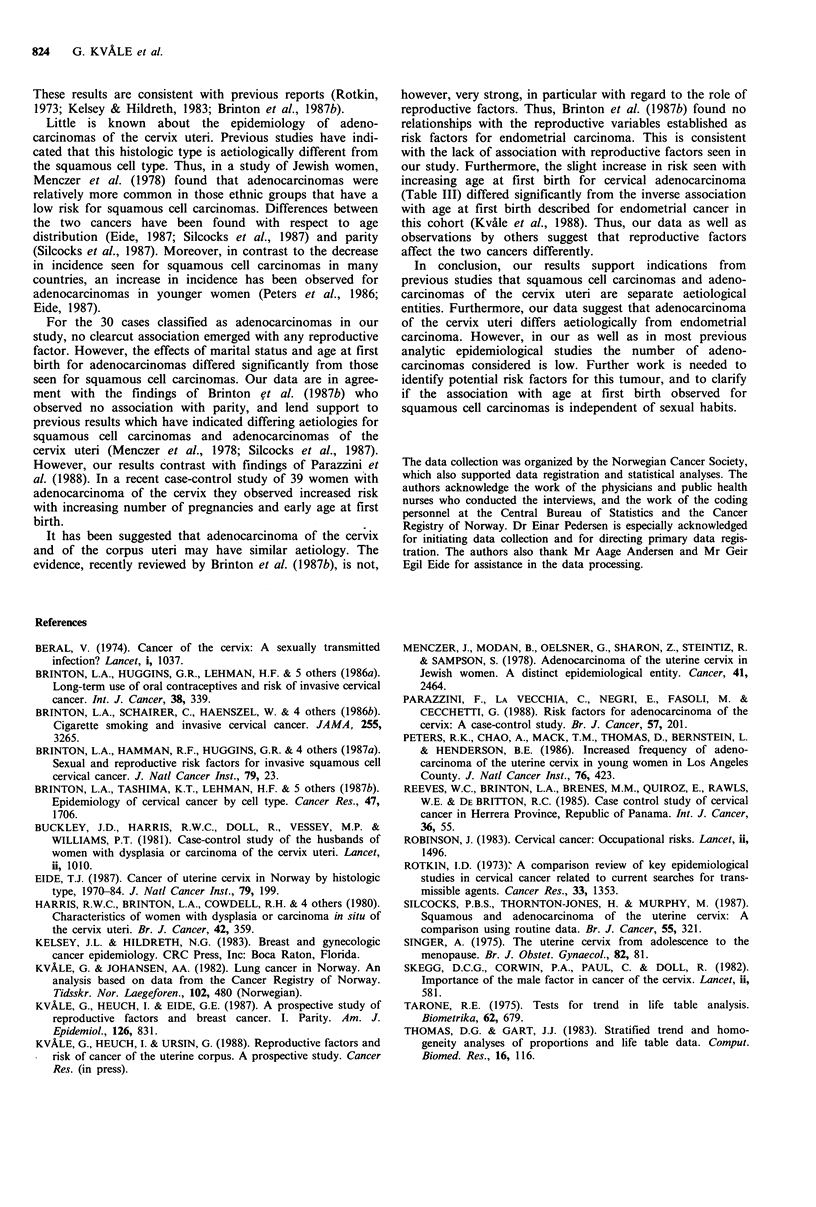# Reproductive factors and risk of cervical cancer by cell type. A prospective study.

**DOI:** 10.1038/bjc.1988.318

**Published:** 1988-12

**Authors:** G. Kvåle, I. Heuch, S. Nilssen

**Affiliations:** Department of Hygiene and Social Medicine, University of Bergen, Norway.

## Abstract

Relationships between reproductive variables and risk of cervical cancer were examined in a follow-up of 62,079 women in Norway from 1961 through 1980. For the 342 cases classified as squamous cell carcinomas, a higher risk was observed in ever married than in never married women. The risk was especially high among women married more than once and women who were widowed or divorced before start of follow-up. High age at first birth was associated with low risk. The estimated odds ratio for women with first birth at age 35 years or later versus 19 years or earlier was 0.18 (P less than 0.001) in analyses with adjustment for age, urban-rural place of residence and parity. In analyses with adjustment for age at first birth, neither parity or age at first marriage, nor age at menarche or menopause showed significant associations with squamous cell carcinoma. For the 30 cases classified as adenocarcinomas, no significant associations emerged with reproductive factors. The effects of marital status as well as age at first birth differed significantly between adenocarcinomas and squamous cell carcinomas, suggesting dissimilar aetiologies. Although associations between reproductive factors and squamous cell carcinoma may largely be secondary to relationships with sexual habits, there are indications that the association with age at first birth cannot be entirely explained in this way.


*Br. J. Cancer* (1988), **58**, 820–824 © The Macmillan Press Ltd., 1988

### Reproductive factors and risk of cervical cancer by cell type. A prospective study

G. Kvåle[1], I. Heuch[2] & S. Nilssen[1]

[1]*Department of Hygiene and Social Medicine, and* [2]*Department of Mathematics, University of Bergen, Norway.*

**Summary** Relationships between reproductive variables and risk of cervical cancer were examined in a follow-up of 62,079 women in Norway from 1961 through 1980. For the 342 cases classified as squamous cell carcinomas, a higher risk was observed in ever married than in never married women. The risk was especially high among women married more than once and women who were widowed or divorced before start of follow-up. High age at first birth was associated with low risk. The estimated odds ratio for women with first birth at age 35 years or later *versus* 19 years or earlier was 0.18 ($P<0.001$) in analyses with adjustment for age, urban-rural place of residence and parity. In analyses with adjustment for age at first birth, neither parity or age at first marriage, nor age at menarche or menopause showed significant associations with squamous cell carcinoma. For the 30 cases classified as adenocarcinomas, no significant associations emerged with reproductive factors. The effects of marital status as well as age at first birth differed significantly between adenocarcinomas and squamous cell carcinomas, suggesting dissimilar aetiologies. Although associations between reproductive factors and squamous cell carcinoma may largely be secondary to relationships with sexual habits, there are indications that the association with age at first birth cannot be entirely explained in this way.

Observed relationships between reproductive factors and cervical cancer have generally been regarded as secondary to associations with sexual habits (Kelsey & Hildreth, 1983). However, in a recent case-control study (Brinton *et al.*, 1987*a*) the effect of the number of births was not eliminated by control for age at first intercourse or number of sexual partners. Furthermore, both that study (Brinton *et al.*, 1987*b*) and recent reports on trends in incidence (Peters *et al.*, 1986; Eide, 1987) indicate that adenocarcinomas of the cervix uteri may differ aetiologically from the squamous cell carcinomas.

Within the context of a large prospective study of cancer in women, we have evaluated the importance of reproductive variables as risk factors for cancer of the cervix uteri. In particular, we have studied differences between invasive tumours of the squamous cell type and adenocarcinomas.

### Materials and methods

In connection with a screening programme for breast cancer in Norway in 1956–1959, detailed data on reproductive factors were collected through personal interviews. The cohort and the methods of follow-up and statistical analysis have been described previously (Kvåle *et al.*, 1987). Of 85,063 women aged 32–74 years by January 1, 1961 in the three counties of Nord-Trøndelag, Aust-Agder and Vestfold, 63,090 women attended the screening programme and were interviewed. After exclusion of 1,011 participants who reported hysterectomy or therapeutic radiation of the genital organs, 62,079 women remained for follow-up.

The official registration number served as a unique identification of the record for each woman and was used to link follow-up information to our files. Complete information concerning emigrations and deaths was obtained from the Central Bureau of Statistics. Data on cancer registrations, including date of diagnosis and histological type, were supplied by the Cancer Registry of Norway.

A total of 392 cases classed as invasive cancer of the cervix uteri (ICD 7th Revision, 171) were diagnosed in the cohort during the period of follow-up, from January 1, 1961 through 1980. The diagnosis was supported by a histological examination of a surgical specimen from the primary tumour and/or by autopsy in 387 cases (98.7%). Of these, 342 were classified as squamous cell carcinomas, 30 as adenocarcinomas, 3 as adenosquamous carcinomas and 12 as carcinomas, not otherwise specified. Separate analyses were carried out for the squamous cell carcinomas and the adenocarcinomas. The analyses were adjusted for age at start of follow-up (with 5-year age groups) and urban/rural place of residence, and in special cases other demographic and reproductive variables. The adjustment was made by forming a stratum for each combination of covariables. Stratified logistic regression analyses were carried out according to the procedure described by Thomas & Gart (1983).

Of the 62,079 participants included, 13,828 died and 124 emigrated in the period 1961–1980. In the estimation procedure, a correction for death and emigration was introduced by decreasing the initial number at risk by half the number of such events occurring among those who did not develop cervical cancer. The expected numbers of cases at the various levels of the study variable under the hypothesis of no association were derived in analyses adjusted for times until censoring (Tarone, 1975). These analyses also produced two-tailed $P$-values for linear trend. Because of missing values for certain reproductive variables, the number of cases varied somewhat between analyses.

### Results

Table I shows that squamous cell carcinomas were slightly more common in urban areas and in the county of Nord-Trøndelag, although differences according to place of residence were not statistically significant. However, significant differences in risk were observed between occupational categories ($P<0.001$), with the highest risk in the group 'fishing, ship officers, crew'. The number of adenocarcinomas was small in each subgroup, and indicated risk differences were not statistically significant.

For squamous cell carcinomas a strong effect of marital status was observed, with particularly high risk estimates in women married more than once, and in those who were widowed or divorced at the time of interview (Table II). These associations obtained in nulliparous as well as in parous women. For adenocarcinomas no such trend was indicated, the risk being highest in the never married group. The distribution of cases in the never and ever married categories differed significantly from that observed for the squamous cell carcinomas ($P=0.05$).

Correspondence: G. Kvåle
Received: 23 February 1988; and in revised form 18 July 1988.

**Table I** Distribution of respondents and cases of cervical carcinoma; and observed/expected ratio (O/E) by demographic variables, prospective study, Norway, 1961–1980.[a]

| | | *Squamous cell carcinoma* | | *Adenocarcinoma* | |
|---|---|---|---|---|---|
| | *Respondents* | *O* | *O/E* | *O* | *O/E* |
| Total series | 62,079 | 342 | 1.00 | 30 | 1.00 |
| Place of residence: | | | | | |
| Urban | 13,080 | 84 | 1.17 | 5 | 0.78 |
| Rural | 48,999 | 258 | 0.96 | 25 | 1.06 |
| County: | | | | | |
| Vestfold | 27,151 | 150 | 0.99 | 14 | 1.05 |
| Aust-Agder | 13,576 | 68 | 0.93 | 4 | 0.61 |
| Nord-Trøndelag | 21,352 | 124 | 1.06 | 12 | 1.18 |
| Occupational category[b]: | | | | | |
| Professional, private enterprise | 9,641 | 45 | 0.84 | 3 | 0.65 |
| Clerical work | 7,598 | 29 | 0.65 | 3 | 0.79 |
| Fishing, ship officers, crew | 5,273 | 46 | 1.56 | 2 | 0.79 |
| Farm and forestry work | 13,474 | 56 | 0.79 | 8 | 1.23 |
| Industrial work | 7,656 | 44 | 0.99 | 6 | 1.66 |
| Domestic and other work | 10,936 | 74 | 1.23 | 8 | 1.49 |
| Not specified | 7,501 | 48 | 1.24 | 0 | – |

[a]Adjusted for age at start of follow-up; [b]Own or husband's occupation.

**Table II** Cervical carcinoma by marital status. Observed (O) and expected (E) number of cases and relative odds estimate (R) by cell type[a].

| *Marital status at time of interview* | *Squamous cell carcinoma* | | | *Adenocarcinoma* | | |
|---|---|---|---|---|---|---|
| | *O* | *E* | *R*[b] | *O* | *E* | *R*[b] |
| Never married | 16 | 34.0 | 1.0 | 4 | 2.9 | 1.0 |
| Married, no previous marriage | 283 | 281.7 | 2.1** | 24 | 23.2 | 0.8 |
| Married, one or more previous marriages | 10 | 4.2 | 5.6*** | 0 | 0.4 | – |
| Widowed | 20 | 12.7 | 3.8*** | 0 | 1.3 | – |
| Divorced | 6 | 2.4 | 5.7*** | 0 | 0.2 | – |

[a]Among women with known parity and marital status, adjusted for age at start of follow-up and urban-rural place of residence; [b]Odds estimate (R) relative to never married women.
Two-tailed $P$-values: **$0.001 < P \leq 0.01$; ***$P \leq 0.001$.

The risk of squamous cell carcinoma was significantly higher in parous than in nulliparous women (Table III). A similar but weaker and non-significant association was observed for adenocarcinomas. An apparent increase in risk with increasing parity for the squamous cell carcinomas observed in initial analyses, was not seen after adjustment for age at first birth. Early first birth was associated with high risk for squamous cell carcinomas, but not for adenocarcinomas. The difference between histologic types was statistically significant ($P = 0.02$). The association with age at first birth for squamous cell carcinoma remained after adjustment for parity and age at last birth, and was consistently found in all subgroups according to demographic and reproductive variables. An association between high age at last birth and low risk of squamous cell carcinoma seen in initial analyses was, however, accounted for by age at first birth (Table III).

Low age at first marriage was associated with increased risk of squamous cell carcinoma. However, the association was weak and not statistically significant. The questionnaire elicited information on year, but not exact date, of first marriage and first and last birth. No association with age at marriage remained in analyses with adjustment for age at first birth among women with their first delivery after the year of first marriage (Table IV). Women who had given birth before the year of marriage were at particularly high risk. In this group, with 36 cases of squamous cell carcinoma, the odds estimate relative to women with their first delivery after the year of marriage was 3.33 (95% CI 2.27–4.05). Those who delivered in the same calendar year as their first marriage took place were at intermediate risk, with an odds estimate relative to women with first delivery after the year of first marriage of 1.81 (75 cases, 95% CI 1.35–2.43).

Age at menarche and menopause were neither associated with risk of squamous cell carcinoma nor adenocarcinoma (Table V). The number of abortions showed a non-significant positive association with squamous cell carcinoma in analysis with adjustment for age, urban-rural place of residence, parity and age at first birth (relative odds: 1.46, 95% CI 0.87–2.45, for women reporting 3 or more abortions versus women reporting none). For adenocarcinoma no association with number of abortions was observed.

### Discussion

In agreement with previous reports (Kelsey & Hildreth, 1983) we have found a high risk of cervical squamous cell carcinoma in ever married women. The risk was especially high in women married more than once and in women who were widowed or divorced before start of follow-up. Associations with such demographic factors have generally been regarded as secondary to more direct associations with sexual habits, involving multiple partners and early age at first intercourse (Rotkin, 1973; Kelsey & Hildreth, 1983).

The significant difference in risk between occupational categories, with the highest risk observed in the group 'fishing, ship officers and crew', is consistent with the contention that the sexual behaviour of the husband is important in the aetiology (Buckley *et al.*, 1981; Skegg *et al.*, 1982). Our finding is in agreement with a report of

**Table III** Cervical carcinoma by parity, age at first and last birth and age at first marriage. Observed (O) and expected (E) number of cases, O/E ratio and relative odds estimate (R) by cell type.

| | *Squamous cell carcinoma* | | | *Adenocarcinoma* | | |
|---|---|---|---|---|---|---|
| | *O* | *E* | *O/E* | *O* | *E* | *O/E* |
| Parity[a]: Nulliparous | 35 | 59.8 | 0.6 | 4 | 5.1 | 0.8 |
| Parous | 304 | 279.2 | 1.1 | 24 | 22.9 | 1.0 |
| R (parous *vs.* nulliparous with 95% CI) | 1.87*** | (1.32–2.66) | | 1.33 | (0.46–3.86) | |
| Parity[b]: 1 | 57 | 51.7 | 1.1 | 6 | 5.6 | 1.1 |
| 2 | 92 | 95.1 | 1.0 | 5 | 7.4 | 0.7 |
| 3 | 71 | 68.1 | 1.0 | 8 | 4.8 | 1.7 |
| 4 | 28 | 36.6 | 0.8 | 3 | 2.5 | 1.2 |
| ≥5 | 39 | 35.5 | 1.1 | 1 | 2.8 | 0.4 |
| R (parity ≥5 *vs.* parity 1 with 95% CI) | 0.90 | (0.60–1.37) | | 0.80 | (0.18–3.58) | |
| Age at first birth (years)[c]: | | | | | | |
| ≤19 | 30 | 16.5 | 1.8 | 0 | 1.0 | 0.0 |
| 20–24 | 131 | 106.4 | 1.2 | 8 | 8.1 | 1.0 |
| 25–29 | 93 | 105.0 | 0.9 | 11 | 8.1 | 1.4 |
| 30–34 | 25 | 42.8 | 0.6 | 0 | 3.7 | 0.0 |
| ≥35 | 8 | 16.3 | 0.5 | 4 | 2.1 | 1.9 |
| R (age at first birth ≥35 *vs.* ≤19 with 95% CI) | 0.18*** | (0.10–0.31) | | 1.60 | (0.27–9.60) | |
| Age at last birth (years)[d]: | | | | | | |
| ≤24 | 11 | 12.2 | 0.9 | 0 | 0.3 | 0.0 |
| 25–29 | 53 | 60.5 | 0.9 | 6 | 3.3 | 1.8 |
| 30–34 | 90 | 83.4 | 1.1 | 6 | 7.7 | 0.8 |
| 35–39 | 53 | 52.6 | 1.0 | 4 | 3.6 | 1.1 |
| ≥40 | 22 | 20.3 | 1.1 | 1 | 2.1 | 0.5 |
| R (age at last birth ≥40 *vs.* ≤24 with 95% CI) | 1.61 | (0.78–3.33) | | 0.16 | (0.01–2.55) | |
| Age at first marriage (years)[e]: | | | | | | |
| ≤19 | 26 | 18.1 | 1.4 | 1 | 1.3 | 0.8 |
| 20–24 | 111 | 116.4 | 1.0 | 8 | 8.6 | 0.9 |
| 25–29 | 95 | 90.4 | 1.1 | 6 | 7.1 | 0.8 |
| 30–34 | 25 | 28.8 | 0.9 | 4 | 3.0 | 1.3 |
| ≥35 | 9 | 12.3 | 0.7 | 3 | 2.0 | 1.5 |
| R (age at first marriage ≥35 *vs.* ≤19 with 95% CI) | 0.63 | (0.35–1.14) | | 2.40 | (0.40–14.3) | |

[a]Among women with known parity, adjusted for age at start of follow-up and urban-rural place of residence; [b]Among parous women with known parity and age at first birth, adjusted for age at start of follow-up, urban-rural place of residence and age at first birth; [c]Among women with known parity and age at first birth, adjusted for age at start of follow-up, urban-rural place of residence and parity; [d]Among parous women with ≥2 births, adjusted for age at start of follow-up, urban-rural place of residence, parity and age at first birth; [e]Among women with known parity and age at first marriage, adjusted for age at start of follow-up, urban-rural place of residence and parity.
Two-tailed $P$-values: ***$P \leq 0.001$.

**Table IV** Squamous cell carcinoma of the cervix uteri by age at first birth and age at first marriage among women with year of first birth later than year of first marriage. Observed and expected number of cases (O/E), and relative odds estimate (R) for age at first birth and age at first marriage[a].

| *Age at first birth or first marriage (years)* | | *≤19* | *20–24* | *25–29* | *30–34* | *≥35* | *Total* | *R (95% CI)* |
|---|---|---|---|---|---|---|---|---|
| Age at first birth: | | | | | | | | |
| Adjusted for age, urban-rural place of residence and parity | O/E | 0/1.9 | 52/37.2 | 57/54.9 | 15/24.5 | 4/9.5 | 128 | 0.20*** (0.08–0.49) |
| Also adjusted for age at first marriage, marital status and occupational group[b] | O/E | 0/2.2 | 50/42.5 | 54/54.7 | 14/17.9 | 2/2.8 | 120 | 0.26 (0.05–1.26) |
| Age at first marriage: | | | | | | | | |
| Adjusted for age, urban-rural place of residence and parity | O/E | 8/6.7 | 65/56.2 | 46/46.8 | 8/14.0 | 1/4.3 | 128 | 0.28** (0.11–0.70) |
| Also adjusted for age at first birth, marital status and occupational group[c] | O/E | 8/7.5 | 57/60.6 | 45/41.3 | 7/7.4 | 1/1.3 | 118 | 1.40 (0.28–6.91) |

[a]Relative odds estimate (R) for age at first birth or age at first marriage ≥35 years *vs.* ≤19, based on logistic regression analyses with 5 levels of age at first birth and age at first marriage; [b]Eight cases in strata with all respondents in the same category for age at first birth were excluded from the analysis; [c]Ten cases in strata with all respondents in the same category for age at first marriage was excluded from the analysis.
Two tailed $P$-values for trend: ** $0.001 < P \leq 0.01$; ***$P \leq 0.001$.

**Table V** Cervical carcinoma by menarche and menopause. Observed (O) and expected (E) number of cases, O/E ratio and relative odds estimate (R), by cell type[a].

| | *Squamous cell carcinoma* | | | *Adenocarcinoma* | | |
|---|---|---|---|---|---|---|
| | *O* | *E* | *O/E* | *O* | *E* | *O/E* |
| Age at menarche[b]: | | | | | | |
| ≤12 | 51 | 36.0 | 1.4 | 3 | 2.7 | 1.1 |
| 13 | 60 | 65.0 | 0.9 | 6 | 5.1 | 1.2 |
| 14 | 97 | 109.4 | 0.9 | 10 | 9.0 | 1.1 |
| 15 | 77 | 71.0 | 1.1 | 6 | 6.4 | 0.9 |
| 16 | 26 | 30.8 | 0.8 | 3 | 3.0 | 1.0 |
| ≥17 | 19 | 17.8 | 1.1 | 0 | 1.8 | – |
| R (age at menarche ≥17 *vs.* ≤12 with 95% CI) | 0.79 | (0.52–1.21) | | 0.45 | (0.10–1.95) | |
| Age at menopause[c]: | | | | | | |
| ≤45 | 17 | 15.9 | 1.1 | 1 | 2.1 | 0.5 |
| 46–47 | 10 | 9.2 | 1.1 | 3 | 1.2 | 2.4 |
| 48–49 | 14 | 14.7 | 1.0 | 3 | 2.4 | 1.3 |
| 50–51 | 17 | 19.4 | 0.9 | 3 | 3.5 | 0.9 |
| 52–53 | 13 | 12.0 | 1.1 | 1 | 2.1 | 0.5 |
| ≥54 | 6 | 5.8 | 1.0 | 1 | 0.7 | 1.4 |
| R (age at menopause ≥54 *vs.* ≤45 with 95% CI) | 0.92 | (0.43–1.96) | | 0.77 | (0.11–5.17) | |

[a]Relative odds estimate based on logistic regression analyses with 6 levels of age at menarche and age at menopause respectively; [b]Among women with known parity and age at menarche, adjusted for age at start of follow-up, urban-rural place of residence and parity; [c]Among women with known parity and age at menopause, adjusted for age at start of follow-up, urban-rural place of residence and parity.

standardized mortality ratios (SMRs) for cervical cancer in England and Wales by husband's occupation (Beral, 1974). Here the highest SMRs were observed among wives of deckhands, barge and boatmen (SMR = 257) and wives of fishermen (SMR = 263). These occupations, which are characterized by longlasting absence from home, may be associated with a higher frequency of extramarital sexual relationships. However, other explanations have also been proposed (Robinson, 1983).

Among the reproductive factors studied, only low age at first birth was significantly associated with high risk. In our study no information was available on sexual habits and other potential confounders like use of oral contraceptives (Brinton *et al.*, 1986*a*) and cigarette smoking (Brinton *et al.*, 1986*b*). As discussed previously (Kvåle *et al.*, 1987), oral contraceptives should not be an important confounder, as a majority of the women in our cohort were postmenopausal when oral contraceptives were introduced in Norway. Furthermore, use of oral contraceptives is probably associated with a late rather than an early first birth, and such confounding, if present, would tend to mask an inverse relationship with age at first birth. Cigarette smoking might be more prevalent among women with an early first delivery. However, it seems unlikely that an association between cigarette smoking and reproductive factors should be of a magnitude explaining the association with age at first birth seen in our study. Furthermore, from what is known about smoking-habits among women in Norway in different cohorts (Kvåle & Johansen, 1982), a large majority of the women in our study should be non-smokers.

There is abundant evidence that sexual habits are the major determinants of risk of squamous cell carcinoma of the cervix uteri (Rotkin, 1973; Brinton *et al.*, 1987*a*). Thus, the inverse association with age at first birth might be secondary to an association with early age at first intercourse or a promiscuous behaviour involving multiple sexual partners. Both age at first birth and age at first marriage are most likely associated with age at first intercourse. However, among women with their first birth after the year of marriage, age at first marriage should show the closer association. The lack of association with age at first marriage in this group, after adjustment for age at first birth, occupation and marital status (Table IV), suggests that early age at first intercourse may not be linked to an increased risk. In a recent report showing a strong and linear relationship with the number of sexual partners, early first intercourse was significantly, although not linearly, associated with risk (Brinton *et al.*, 1987*a*). A previous study showed no clear relationship of risk to age at first intercourse (Reeves *et al.*, 1985). Considering *in situ* carcinomas, Reeves *et al.* (1985) observed a significant association, whereas Harries *et al.* (1980) found no independent effect of early intercourse after adjustment for number of sexual partners.

The relatively strong inverse association with age at first birth seen in this study, in analyses with adjustment for marital status, occupation and age at first marriage (Table IV), suggests that pregnancy-related factors other than sexual habits may be of aetiological importance. Thus, studying pregnant women, Singer (1975) observed increased frequency and size of squamous epithelial metaplasia of the columnar epithelium near the squamocolumnar junction of the cervix, particularly during the first pregnancy. As a result of an eversion of the endocervix during the first pregnancy (Singer, 1975), the transformation zone of columnar epithelium increases in size and becomes more exposed to the acidic vaginal secretions. This may increase the susceptibility of the cervix to sexually transmitted infections and stimulate metaplasia of the columnar epithelium.

In our study, the number of births showed no effect independent of age at first birth. In a recent study, Brinton *et al.* (1987*a*) observed that number of births was positively and age at first birth inversely associated with risk of squamous cell carcinoma in analyses with adjustment for age at first intercourse, number of sexual partners and several other potential confounders. However, the association with age at first birth was not statistically significant in their study, although risk estimates were similar to those observed by us. The lack of independent association with number of births seen by us is consistent with findings in several previous studies (Rotkin, 1973; Kelsey & Hildreth, 1983). In studies where increased risk has been observed in multiparous women, age at first birth has generally not been adjusted for. Thus, although our data suggest that factors related to the first pregnancy may be of importance, it seems less likely that factors associated with every pregnancy influence the risk of squamous cell cervical carcinoma.

In analysis with adjustment for age at first birth, neither age at last birth nor age at menarche or menopause showed significant associations with risk of squamous cell carcinoma.

These results are consistent with previous reports (Rotkin, 1973; Kelsey & Hildreth, 1983; Brinton et al., 1987b).

Little is known about the epidemiology of adenocarcinomas of the cervix uteri. Previous studies have indicated that this histologic type is aetiologically different from the squamous cell type. Thus, in a study of Jewish women, Menczer et al. (1978) found that adenocarcinomas were relatively more common in those ethnic groups that have a low risk for squamous cell carcinomas. Differences between the two cancers have been found with respect to age distribution (Eide, 1987; Silcocks et al., 1987) and parity (Silcocks et al., 1987). Moreover, in contrast to the decrease in incidence seen for squamous cell carcinomas in many countries, an increase in incidence has been observed for adenocarcinomas in younger women (Peters et al., 1986; Eide, 1987).

For the 30 cases classified as adenocarcinomas in our study, no clearcut association emerged with any reproductive factor. However, the effects of marital status and age at first birth for adenocarcinomas differed significantly from those seen for squamous cell carcinomas. Our data are in agreement with the findings of Brinton et al. (1987b) who observed no association with parity, and lend support to previous results which have indicated differing aetiologies for squamous cell carcinomas and adenocarcinomas of the cervix uteri (Menczer et al., 1978; Silcocks et al., 1987). However, our results contrast with findings of Parazzini et al. (1988). In a recent case-control study of 39 women with adenocarcinoma of the cervix they observed increased risk with increasing number of pregnancies and early age at first birth.

It has been suggested that adenocarcinoma of the cervix and of the corpus uteri may have similar aetiology. The evidence, recently reviewed by Brinton et al. (1987b), is not, however, very strong, in particular with regard to the role of reproductive factors. Thus, Brinton et al. (1987b) found no relationships with the reproductive variables established as risk factors for endometrial carcinoma. This is consistent with the lack of association with reproductive factors seen in our study. Furthermore, the slight increase in risk seen with increasing age at first birth for cervical adenocarcinoma (Table III) differed significantly from the inverse association with age at first birth described for endometrial cancer in this cohort (Kvåle et al., 1988). Thus, our data as well as observations by others suggest that reproductive factors affect the two cancers differently.

In conclusion, our results support indications from previous studies that squamous cell carcinomas and adenocarcinomas of the cervix uteri are separate aetiological entities. Furthermore, our data suggest that adenocarcinoma of the cervix uteri differs aetiologically from endometrial carcinoma. However, in our as well as in most previous analytic epidemiological studies the number of adenocarcinomas considered is low. Further work is needed to identify potential risk factors for this tumour, and to clarify if the association with age at first birth observed for squamous cell carcinomas is independent of sexual habits.

The data collection was organized by the Norwegian Cancer Society, which also supported data registration and statistical analyses. The authors acknowledge the work of the physicians and public health nurses who conducted the interviews, and the work of the coding personnel at the Central Bureau of Statistics and the Cancer Registry of Norway. Dr Einar Pedersen is especially acknowledged for initiating data collection and for directing primary data registration. The authors also thank Mr Aage Andersen and Mr Geir Egil Eide for assistance in the data processing.

### References

BERAL, V. (1974). Cancer of the cervix: A sexually transmitted infection? *Lancet*, **i**, 1037.

BRINTON, L.A., HUGGINS, G.R., LEHMAN, H.F. & 5 others (1986a). Long-term use of oral contraceptives and risk of invasive cervical cancer. *Int. J. Cancer*, **38**, 339.

BRINTON, L.A., SCHAIRER, C., HAENSZEL, W. & 4 others (1986b). Cigarette smoking and invasive cervical cancer. *JAMA*, **255**, 3265.

BRINTON, L.A., HAMMAN, R.F., HUGGINS, G.R. & 4 others (1987a). Sexual and reproductive risk factors for invasive squamous cell cervical cancer. *J. Natl Cancer Inst.*, **79**, 23.

BRINTON, L.A., TASHIMA, K.T., LEHMAN, H.F. & 5 others (1987b). Epidemiology of cervical cancer by cell type. *Cancer Res.*, **47**, 1706.

BUCKLEY, J.D., HARRIS, R.W.C., DOLL, R., VESSEY, M.P. & WILLIAMS, P.T. (1981). Case-control study of the husbands of women with dysplasia or carcinoma of the cervix uteri. *Lancet*, **ii**, 1010.

EIDE, T.J. (1987). Cancer of uterine cervix in Norway by histologic type, 1970–84. *J. Natl Cancer Inst.*, **79**, 199.

HARRIS, R.W.C., BRINTON, L.A., COWDELL, R.H. & 4 others (1980). Characteristics of women with dysplasia or carcinoma *in situ* of the cervix uteri. *Br. J. Cancer*, **42**, 359.

KELSEY, J.L. & HILDRETH, N.G. (1983). Breast and gynecologic cancer epidemiology. CRC Press, Inc: Boca Raton, Florida.

KVÅLE, G. & JOHANSEN, AA. (1982). Lung cancer in Norway. An analysis based on data from the Cancer Registry of Norway. *Tidsskr. Nor. Laegeforen.*, **102**, 480 (Norwegian).

KVÅLE, G., HEUCH, I. & EIDE, G.E. (1987). A prospective study of reproductive factors and breast cancer. I. Parity. *Am. J. Epidemiol.*, **126**, 831.

KVÅLE, G., HEUCH, I. & URSIN, G. (1988). Reproductive factors and risk of cancer of the uterine corpus. A prospective study. *Cancer Res.* (in press).

MENCZER, J., MODAN, B., OELSNER, G., SHARON, Z., STEINTIZ, R. & SAMPSON, S. (1978). Adenocarcinoma of the uterine cervix in Jewish women. A distinct epidemiological entity. *Cancer*, **41**, 2464.

PARAZZINI, F., LA VECCHIA, C., NEGRI, E., FASOLI, M. & CECCHETTI, G. (1988). Risk factors for adenocarcinoma of the cervix: A case-control study. *Br. J. Cancer*, **57**, 201.

PETERS, R.K., CHAO, A., MACK, T.M., THOMAS, D., BERNSTEIN, L. & HENDERSON, B.E. (1986). Increased frequency of adenocarcinoma of the uterine cervix in young women in Los Angeles County. *J. Natl Cancer Inst.*, **76**, 423.

REEVES, W.C., BRINTON, L.A., BRENES, M.M., QUIROZ, E., RAWLS, W.E. & DE BRITTON, R.C. (1985). Case control study of cervical cancer in Herrera Province, Republic of Panama. *Int. J. Cancer*, **36**, 55.

ROBINSON, J. (1983). Cervical cancer: Occupational risks. *Lancet*, **ii**, 1496.

ROTKIN, I.D. (1973). A comparison review of key epidemiological studies in cervical cancer related to current searches for transmissible agents. *Cancer Res.*, **33**, 1353.

SILCOCKS, P.B.S., THORNTON-JONES, H. & MURPHY, M. (1987). Squamous and adenocarcinoma of the uterine cervix: A comparison using routine data. *Br. J. Cancer*, **55**, 321.

SINGER, A. (1975). The uterine cervix from adolescence to the menopause. *Br. J. Obstet. Gynaecol.*, **82**, 81.

SKEGG, D.C.G., CORWIN, P.A., PAUL, C. & DOLL, R. (1982). Importance of the male factor in cancer of the cervix. *Lancet*, **ii**, 581.

TARONE, R.E. (1975). Tests for trend in life table analysis. *Biometrika*, **62**, 679.

THOMAS, D.G. & GART, J.J. (1983). Stratified trend and homogeneity analyses of proportions and life table data. *Comput. Biomed. Res.*, **16**, 116.